# Key Molecules of Fatty Acid Metabolism in Gastric Cancer

**DOI:** 10.3390/biom12050706

**Published:** 2022-05-15

**Authors:** Chunlei Li, Lilong Zhang, Zhendong Qiu, Wenhong Deng, Weixing Wang

**Affiliations:** Department of General Surgery, Renmin Hospital of Wuhan University, Wuhan 430060, China; 2015302180080@whu.edu.cn (C.L.); dmuzhanglilong@163.com (L.Z.); qcqiuzhendong@whu.edu.cn (Z.Q.)

**Keywords:** gastric cancer, fatty acids, metabolism, key enzymes, treatment, prognosis

## Abstract

Fatty acid metabolism is closely linked to the progression of gastric cancer (GC), a very aggressive and life-threatening tumor. This study examines linked molecules, such as Sterol Regulatory Element-Binding Protein 1 (SREBP1), ATP Citrate Lyase (ACLY), Acetyl-CoA Synthases (ACSs), Acetyl-CoA Carboxylase (ACC), Fatty Acid Synthase (FASN), Stearoyl-CoA Desaturase 1 (SCD1), CD36, Fatty Acid Binding Proteins (FABPs), and Carnitine palmitoyltransferase 1 (CPT1), as well as their latest studies and findings in gastric cancer to unveil its core mechanism. The major enzymes of fatty acid de novo synthesis are ACLY, ACSs, ACC, FASN, and SCD1, while SREBP1 is the upstream molecule of fatty acid anabolism. Fatty acid absorption is mediated by CD36 and FABPs, and fatty acid catabolism is mediated by CPT1. If at all possible, we will discover novel links between fatty acid metabolism and a prospective gastric cancer target.

## 1. Introduction

Gastric cancer (GC) is a highly heterogeneous and aggressive malignancy that affects people all over the world. According to recent data, GC is the fifth most common malignancy and the fourth most deadly among tumors, with an estimated one million new cases each year [1,2]. Unfortunately, due to a lack of early diagnosis, a large number of patients, approximately 768,000, died in 2020 all over the world [3]. Surgery, radiotherapy, targeted molecular therapy, and other treatments for GC are used in clinical practice. However, there is still some ambiguity about the inner mechanism, particularly when it comes to the metabolic mechanism and cancer. The metabolic profile of malignant tumors is increasingly being described, which is encouraging. According to Reinfeld et al., glucose is allocated to infiltrating immune cells, rather than cancer cells, while glutamine and lipids are primarily consumed by cancer cells [4]. According to some researchers, lipid metabolism is one of the three primary energy metabolisms of cells, lipid reprogramming is one of the characteristics of cancer, and fatty acid metabolism is a critical aspect of lipid metabolism [5,6]. Lipid catabolism, as we know, produces a lot of fatty acids, which are necessary for cancer cells to survive. Cancer cells are increasingly reliant on de novo lipogenesis (DNL) and exogenous Fatty acids (FAs) uptake, not only to provide the energy they require for rapid proliferation, but also to support oncogenic signals that promote tumorigenesis and cancer progression [7,8]. As a result, the focus of this review will be on recent developments in the relationship between essential molecules in fatty acid metabolism and GC, as well as the impact of fatty acid synthesis and catabolism on GC development, progression, metastasis, and treatment.

## 2. Functions of Fatty Acids

FAs are the main building blocks of a variety of lipid species (including phospholipids, sphingolipids, and triglycerides), and they are made up of a carboxylic acid group and a hydrocarbon chain with various carbon lengths and desaturation [7,9]. FAs are found in a variety of cell structures and govern a variety of biochemical activities in normal cells, including the creation and regulation of biological membrane fluidity, acting as second messengers in signaling pathways, and serving as a type of energy storage in animals [10,11]. Endogenous fatty acid production relies mostly on DNL, with acetyl-coenzyme A (acetyl-CoA) as the principal substrate, while exogenous fatty acid intake necessitates associated transport molecules, such as CD36 and Fatty Acid Binding Proteins (FABPs) [7].

To maintain normal life processes, the body requires saturated fatty acids (SFAs) and unsaturated fatty acids, which can be obtained by DNL and fatty acid uptake, with unsaturated fatty acids, such as alpha-linolenic acid and linoleic acid, requiring external diet [12]. SFAs are critical energy sources for the body and a crucial component of cellular membrane fluidity, as they are the primary building blocks of phospholipids and cholesterol. SFAs are also used to produce second messenger precursors and are the starting material for intracellular conversion to unsaturated fatty acids and derivatives. Unsaturated fatty acids have been found to increase nerve cell formation, healthy vision, lipid deposits reduction, and immunity enhancement. Oleic acid (OA) is a common monounsaturated fatty acid (MUFA), and arachidonic acid (AA), eicosapentaenoic acid (EPA), and docosahexaenoic acid (DHA) are common polyunsaturated fatty acids that reduce inflammation and are thought to lower the risk of breast and other cancers [8,10,11,13]. By raising the ratio of SFAs to MUFAs in membrane lipids, cancer cells might suppress polyunsaturated fatty acid (PUFA) lipid peroxidation under reactive oxygen species (ROS) conditions, allowing sufficient fluidity of membrane production and thereby enhancing tumor cell motility and invasion [14,15,16].

FAs are crucial for tumor cells, in addition to normal cells, since they maintain membrane biosynthesis during fast proliferation and provide a critical energy source in metabolically stressed settings. FAs and fatty acid metabolites have been implicated in cancer formation and progression in recent years, and the interaction between fatty acid metabolism and cancer is becoming more complex as research advances. We also report the most recent findings in GC on molecules relevant to fatty acid metabolism.

## 3. Key Molecules in Fatty Acid Metabolism

### 3.1. Key Molecules in the Endogenous Synthesis of FAs

#### 3.1.1. Sterol Regulatory Element-Binding Protein 1 (SREBP1) 

SREBP1, also called sterol regulatory element-binding transcription factor 1 (SREBF1), belongs to the bHLH-Zip family and contains two isoforms, SREBP1A and SREBP1C, which are transcription factors that regulate mammalian lipid metabolism [17]. SREBP1 regulates both fatty acid synthesis and cholesterol synthesis by regulating the expression of genes related to lipid synthesis [18,19]. For example, SREBP1 can bind to the promoters of ATP Citrate Lyase (ACLY) and Acyl-CoA Synthetase Short Chain Family Member 2 (ACSS2) to regulate lipid metabolism at the transcriptional level, and SREBP1 also affects the expression of Acetyl-CoA Carboxylase (ACC), Fatty Acid Synthase (FASN), and Stearoyl-CoA Desaturase 1 (SCD1) [20,21,22]. SREBP1C expression is increased in GC tissues, and the stimulation of hypoxia-inducible factor 1α (HIF-1α) increases the expression of SREBP1C and FASN genes [23,24]. In a study by Sun et al., the activation of SREBP-1C in GC resulted in the upregulation of SCD1 and FASN and downregulation of ELOVL6. The knockdown of SREBP1C significantly inhibited the proliferation, invasiveness, and migration of GC cells [25]. The advanced GC VEGFR2-targeting drug apatinib regulates glutathione peroxidase 4 (GPX4) transcription in GC cells by mediating the binding of SREBP1A to the GPX4 promoter region, which may account for the effectiveness of apatinib in multi-drug-resistant GC cells [26]. Oregano EO inhibited the lipogenesis pathway by downregulating transcripts and proteins, such as ACC, FASN, SREBP1 (fatty acid biosynthesis), and HMGCR (cholesterol biosynthesis) [27]. SREBP1 has an upstream position in fatty acid synthesis. It regulates the expression of various lipid metabolism genes, and the development of drugs targeting SREBP1 may be a new direction for GC therapy.

#### 3.1.2. ACLY

ACLY is the first key enzyme of DNL [28], and it connects gluconeogenesis and lipid metabolism. Citrate generated by the tricarboxylic acid cycle (TCA) in mitochondria is transferred to the cytoplasm, where it is cleaved into acetyl-CoA and oxaloacetate by ACLY, which is found on the endoplasmic reticulum, linking glucose and fatty acid metabolism [29,30]. ACLY is found on chromosome 17q, which is commonly amplified in GC tissues [31]. Increased copy counts of ACLY [32] were discovered using comparative genomic hybridization on cDNA microarray analysis of DNA copy number alterations. ACLY expression was shown to be considerably higher in GC tissues than in surrounding normal tissues, and it was linked to advanced GC, lymph node metastases, and a shorter survival time [33]. Both FASN and ACLY, which are key enzymes involved in DNL, were increased in the gastric mucosa of *H. pylori*-positive patients compared with *H. pylori*-negative patients [34]. Zheng et al. analyzed 825 survival-related genes in 332 TCGA GC specimens using Cox univariate regression, found 12 mRNAs linked to prognosis, and built a lncRNA-miRNA-mRNA ceRNA network. ACLY is an important upregulated gene [35]. The activity of ACLY is inhibited by high doses of sodium citrate, and intraperitoneal injections of sodium citrate suppressed the growth of GC in mice [36,37]. The overexpression of miR-133b and lncRNA FLJ22763 inhibited GC cell growth and invasion by reducing ACLY expression and activation [38,39]. Cheng et al. used a dual-luciferase reporter gene to evaluate the transcriptional activity of ACLY 3’UTR and found that miR-133b lowered the ACLY transcriptional activity in a PPAR-dependent manner [39]. According to the findings, ACLY overexpression is linked to poor GC outcomes, and targeting ACLY for GC treatment could be a viable option.

#### 3.1.3. Acetyl-CoA Synthases (ACSs)

FAs are converted to acetyl-CoA in the presence of ACSs in cells. The ACS short-chain family (ACSS1–3), the ACS medium-chain family (ACSM1, ACSM2A, ACSM2B, ACSM3–5), the ACS long-chain family (ACSL1 and ACSL3–6), the ACS very long-chain family (ACSVL1–6), the ACS bubblegum family (ACSBG1–2), and the ACS family (ACSF1–4) are subdivided based on substrate specificities toward the chain length of FAs and sequence similarity [40,41]. FAs must be activated by ACSs, which produce fatty acyl-CoA (FA-CoA), before they may reach bioactive pools. ACSS, which consists of Acyl-CoA Synthetase Short Chain Family Member 1 (ACSS1), ACSS2, and Acyl-CoA Synthetase Short Chain Family Member 3 (ACSS3), is responsible for converting acetic acid into acetyl-coenzyme A [42]. The promoter methylation levels of ACSS1 were considerably greater in primary EBV(+) gastric malignancies than in EBV(−) gastric cancers, according to validation [43]. Chang et al. confirmed that ACSS3 is a GC prognostic marker, and that knocking it out might reduce colony formation in regular culture and wound healing under starving conditions. Furthermore, knocking down ACSS3 in GC cells can enhance cell mortality at a base level, and considerably more so under starving conditions [42]. ACSS2 is found in the cytoplasm, and the acetyl-coenzyme A it produces, like ACLY, can be directly engaged in FAs DNL. ACSS2 expression deficit was found to be an independent predictive factor for disease-free survival (DFS) and overall survival (OS) in GC patients in a multifactorial analysis [44]. ACSS2 expression was dramatically increased after ACLY inhibition in research by N. Zaidi et al., showing the relevance of ACSS2 in ACLY disorders [45]. Due to the functional similarity and possible interaction between ACSS2 and ACLY, the study suggests that ACSS2 may act as an accessory pathway to ACLY, and that the simultaneous inhibition of ACLY and ACSS2 is a promising option for inhibiting GC progression and treating GC. Ectopic Acyl-CoA Synthetase Long Chain Family Member 4 (ACSL4) expression decreases cell growth, colony formation, and cell migration in cell-based functional tests, whereas ACSL4 knockdown enhances these effects. ACSL4 knockdown increased the growth of subcutaneous xenografts in vivo in a nude mouse model. Furthermore, Western blot analysis demonstrated that ACSL4 expression had a significant impact on the levels of FAK and P21 proteins. These data imply that ACSL4 suppresses tumor growth in GC [46]. The oncogenicity of MKN01 cells is inhibited by siRNA-mediated Acyl-CoA Synthetase Long Chain Family Member 5 (ACSL5) suppression. Epigenetic changes induce ONECUT2 in intestinal metaplasia, which raises the level of ACSL5, which is also expressed in intestinal metaplasia. As a result, ONECUT2 and ACSL5 may promote intestinal differentiation and GC development in concert [47]. Although ACSs play a similar role in fatty acid metabolism as in gastric cancer, the degree of expression and specific contribution are not the same, according to the findings.

#### 3.1.4. ACC

The rate-limiting enzyme for the synthesis of numerous FAs, including ACC1 and ACC2, is encoded by ACACA, and ACC is a tightly controlled enzyme in the fatty acid production pathway [48]. ACC carboxylates acetyl-CoA to generate malonyl-CoA during the de novo production of Fas [49]. Malonyl-CoA is a direct fatty acid synthesis substrate, as well as a key regulator of fatty acid catabolism. It also plays an important role in fatty acid catabolism [29]. The amount of CD8+ T cell infiltration and immune cytolytic activity (ICA) were significantly and negatively linked with ACC, suggesting that inhibiting ACC improves anti-tumor immunity in GC [24,50,51]. ACC has been demonstrated to be an AMPK target molecule, and AMPK phosphorylation can regulate ACC activity [52]. In GC, the expression of the ACC inactivated form (phosphorylated ACC, pACC) was reduced. The level of pACC was similarly much lower in hypofractionated GC than in highly differentiated GC, implying that hypofractionated GC has enhanced ACC activity [53]. Furthermore, GC patients with a high level of pACC expression had a longer median survival time. Metformin (an AMPK activator)-induced pACC overexpression inhibited GC cell growth and colony formation significantly [51]. According to the findings, pACC downregulation is an important step in gastric carcinogenesis and can predict patient prognosis early on.

#### 3.1.5. FASN

FASN catalyzes the manufacture of endogenous FAs by employing acetyl-CoA as a primer, malonyl coenzyme A as a two-carbon donor, and NADPH as a reducing equivalent to synthesize long-chain FAs [54,55]. FASN transforms excess carbs to FAs, which are esterified to stored lipids by other enzymes and, if needed, supply energy via -oxidation [56]. FASN gene and protein expression is elevated in GC cells under hypoxic culture conditions, according to in vitro studies [23]. The FASN levels are higher in GC patients’ serum and tissues, and FASN and membranous HER2 (mHER2) expression are linked, with patients with concordant FASN and mHER2 expression having worse prognosis [24,57,58,59,60,61,62]. The overexpression of FASN in GC tissues has been linked to shorter survival in bioinformatics studies, and FASN may play a role in tumor formation by controlling macrophage polarization [62]. FASN overexpression was strongly connected with the amount of GC metastases and projected poor prognosis, according to Duan et al., who used immunohistochemistry to detect FASN expression in 167 GC tissues and evaluated the correlation between FASN expression and clinicopathological characteristics [59]. C75 or siFASN, a FASN inhibitor, inhibited endogenous fatty acid metabolism in GC and reduced MACC1 upregulation-induced cell proliferation and oxaliplatin chemo-resistance to various degrees [63]. Orlistat is also a potent FASN inhibitor, and results from a subcutaneous tumor model showed that orlistat treatment inhibited metastatic ability [64]. By targeting the mTOR/Gli1 signaling pathway, FASN inhibition also decreased GC growth and metastasis [60]. FASN may be a promising predictive biomarker for GC patients, and FASN may be a possible target for GC treatment, according to the research above.

#### 3.1.6. SCD1

SCD1 is also a rate-limiting enzyme in the conversion of saturated fatty acids (palmitic acid, PA) to monounsaturated fatty acids (MUFAs) [65,66]. Oleic acid is a typical monounsaturated fatty acid that can be consumed by highly metastatic cancer cells to maintain malignancy in an AMPK-dependent manner [67]. Through the PI3K/Akt signaling pathway, adipocytes can act as an exogenous supply of oleic acid and enhance gastric cancer cell invasion [68]. Many lipids, including phospholipids, triglycerides, and cholesterol esters, are produced using SCD1’s unsaturated FAs as major substrates [69]. SCD1 expression is elevated in GC, and high SCD1 expression in GC patients may indicate poor prognosis [70,71]. SCD1 increases the proliferative and migratory abilities of GC cells, as well as having an anti-iron death impact and speeding up the formation of tumors in mice [71]. In comparison to vehicle-treated mice, the mean tumor volume in the A939572-treated group (an inhibitor of SCD1) was reduced by about 50%. The inhibition of SCD1 caused a delay in tumor growth in a human GC xenograft model [72]. Furthermore, Gao et al. discovered that SCD1 regulates cell stemness via the Hippo/YAP pathway, which promotes gastric carcinogenesis, chemo-resistance, and metastasis, and that knocking down SCD1 makes gastric cancer cells responsive to oxaliplatin [70]. According to the findings, targeted suppression of SCD1 may lower the stemness of GC cells, improving chemosensitivity and avoiding GC metastases.

### 3.2. Key Molecules for Exogenous Uptake of FAs

#### 3.2.1. CD36

CD36 is a scavenger receptor that regulates lipid uptake, immunological recognition, inflammation, molecular adhesion, and death in a variety of cells [73]. CD36 stimulates tumor development and metastasis by allowing cells to take up lipids from the extracellular microenvironment and promoting the oxidation of FAs to create ATP [74,75,76]. CD36 mRNA expression has previously been linked to GC survival, and high CD36 expression has been linked to poor clinicopathological outcomes in GC patients [77,78]. In GC, CD36 upregulation predicts poor prognosis and increases cellular metastasis [79,80]. Palmitic acid promoted GC metastasis via the phosphorylation of AKT, and CD36 promoted GC metastasis as a key mediator mediating AKT/GSK-3/β-catenin signaling, according to Pan et al. [81]. Wang et al. discovered that CD36 mediates c-Myc-induced DEK transcription in GC cells, and that DEK overexpression increases GSK-3/β-catenin signaling [78]. By upregulating the expression of CD36 and Carnitine palmitoyltransferase 1B (CPT1B), IL6, TNF-α, and PITPNC1 improved fatty acid metabolism [82]. CD36 can also operate as a direct target molecule for hydrogen sulfide (H2S), activating long-chain FAs in the cytoplasm and causing lipid metabolic reprogramming [83]. It has also been shown that high fatty acid uptake promotes CD36 transcription and functioning by inducing O-GlcNAcylation glycosylation and activating the NF-B pathway, resulting in GC metastasis, forming a vicious cycle [80]. The findings imply that CD36 plays an important role in GC metastasis, and that targeting CD36 may help to reduce GC metastasis.

#### 3.2.2. FABPs

Most FAs require complexes with certain proteins, such as albumins, lipoproteins, and FABPs, which are classified into nine different types [84]. FABPs bind long-chain FAs and improve fatty acid solubility, according to recent investigations into their structure and function. They have a role in lipid metabolism by facilitating FAs translocation to the cell or mitochondrial membrane [85,86,87,88]. Fatty Acid Binding Protein 1 (FABP1) expression is high and intense in metaplasia and a fraction of gastric adenocarcinomas, although it has no relation to the carcinoma’s development, prognosis, or fatty acid synthase status. The results of Satoh et al. imply that the FABP1 mRNA level is a useful tool for identifying individuals with peritoneal metastasis or at high risk of peritoneal recurrence who might benefit from more potent adjuvant chemotherapy [89,90]. The coexpression of FABP1 and FASN may act as a biomarker for the detection of early GC, according to a quantitative proteomic study of GC tissues [91]. Fatty Acid Binding Protein 3 (FABP3)-negative patients had a greater survival rate than FABP3-positive patients in GC cases [92]. The combination of UPK1B, FABP3, CASP5, and CYP4X1 could predict the result of GC CapeOX treatment, according to a comprehensive analysis [93]. Fatty Acid Binding Protein 4 (FABP4) regulation by a small-molecule FABP4 inhibitor or siFABP4 restores primary cilia, inhibiting the proliferation and migration of GCs, and hence has anticancer potential [94]. According to the GEPIA and UALCAN databases, FABP4 expression was significantly reduced in stomach adenocarcinoma (STAD) tissues, but Western blot results revealed significantly high expression of FABP4 and Fatty Acid Binding Protein 5 (FABP5) in both GC tissues and GC cells. High FABP4 and FABP5 expression is associated with unfavorable pathological characteristics and a poor prognosis in GC [94,95,96,97]. STAT5A-regulated fatty acid metabolism increases the tumorigenic capacity of GC cells in vivo by increasing the expression of FABP5 [98], according to a recent study. The FABP5 gene was silenced, which reduced GC cell invasiveness, stopped cell proliferation, and halted the cell cycle in the G0/G1 phase, resulting in a large increase in apoptosis [95,99]. SBFI-26, an FABP5 inhibitor, may also be a GC candidate drug [95]. PA enters GC cells and enhances FABP5 nuclear transport, which increases the GC nuclear protein levels of SP1, resulting in PA-induced GC metastasis via FABP5/SP1/UCA1 signaling, resulting in effective GC preventative and therapy strategies [100]. FABP4 and FABP5 knockdown in GC cells significantly boosted lipid uptake in tissue-resident memory T cells (TRM) in a tumor cell–T cell co-culture system when compared to FABP4 or FABP5 knockdown alone. Furthermore, knocking down FABP4 or FABP5 in GC cells reduced TRM cell death, and knocking down FABP4 and FABP5 together reduced TRM cell apoptosis even more, demonstrating that GC cells can induce TRM cell apoptosis by depriving TRM cells of lipid uptake [97]. Berberine was reported to decrease GC growth in vitro and in vivo by decreasing the expression of FABP4 and FABP5, resulting in the buildup of FAs and GC cell death in other studies [101]. FABPs have been linked to apoptosis in GC, and the findings show that FABPs may influence apoptosis by controlling cell lipid uptake.

### 3.3. A Key Molecule in Fatty Acid Catabolism

#### Carnitine palmitoyltransferase 1 (CPT1)

The CPT1 family, which includes CPT1A, CPT1B, and CPT1C, is found on the outer mitochondrial membrane and serves as the gatekeeper enzyme for long-chain FAs entrance and subsequent oxidation [102]. Long-chain fatty acids must be transformed to acylcarnitine before entering the mitochondrial matrix for oxidation [103]. Because CPT1 is blocked by malonyl coenzyme A, a component of ACC, fatty acid oxidation is boosted when ACC is suppressed. According to a prior study, pACC can reduce cancer cell proliferation, which could be due to enhanced fatty acid degradation [51,104]. HCP5 targeted miR-3619-5p to upregulate PPARGC1A, which resulted in the transactivation of CPT1 via the PGC1/CEBPB complex and promoted fatty acid oxidation (FAO), according to Wu et al. [105]. CPT1A protein expression was shown to be significantly increased in GC cells and tissues, and was linked to the GC grade, clinical stage, lymph node metastasis, and poor prognosis. CPT1A overexpression promoted the proliferation, invasion, and epithelial–mesenchymal transition (EMT) of GC cells through activating fatty acid oxidation in GC cells by increasing the NADP/NADPH ratio [106,107]. CPT1A succinylated LDHA on K222, which enhanced GC invasion and proliferation by reducing binding and inhibiting LDHA degradation [108]. After fenofibrate treatment, the expression levels of the fatty acid metabolism-related proteins FASN and ACC2 dropped, while the expression levels of p-ACC2 and CPT1A proteins increased significantly [109]. CPT1B was upregulated in oxaliplatin-treated GC cells (HGC27 and MGC803), and the CPT1 inhibitor perhexiline and oxaliplatin inhibited tumor xenograft progression in a subcutaneous xenograft BALB/c nude mouse model of HGC27 cells, suggesting that CPT1-mediated fatty acid translocation and further fatty acid oxidation may be associated with oxaliplatin CPT1C expression was found to be substantially linked to poor DFS and OS in GC patients. Hypoxia-induced CPT1C expression has been linked to a poor prognosis and has been shown to enhance GC cell proliferation [110,111]. These findings imply that CPT1 is a critical enzyme in controlling FAs oxidative catabolism, and that inhibiting GC development and metastasis by lowering CPT1 expression may increase the efficacy of chemotherapeutic drugs.

**Figure 1:**
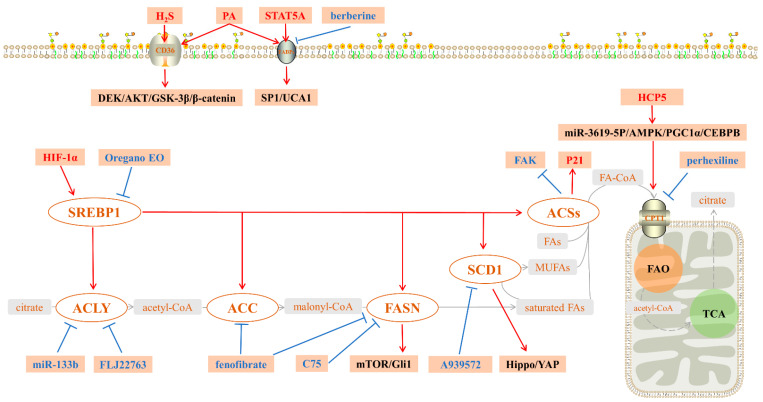
Fatty acid metabolism in GC. Citrate by TCA enters the cytoplasm and is converted to acetyl-CoA by ACLY, then ACC converts acetyl-CoA to malonyl-CoA. Malonyl-CoA is used by FASN as a direct substrate for the DNL to synthesize saturated fatty acids (usually PA). SCD1 is capable of converting saturated fatty acids to MUFAs. ACSs play two main roles in fatty acid metabolism: one is to convert free fatty acids to acetyl-CoA, thus participating in fatty acid synthesis; the other is to convert fatty acids to FA-CoA to be transferred to mitochondria by CPT1 for further oxidative catabolism. SREBP1 regulates the expression of ACLY, ACC, FASN, SCD1, and ACSs at the transcriptional level. CD36 and FABPs are responsible for the uptake of exogenous PA, involved in fatty acid metabolic processes in the cytoplasm. In gastric cancer, FASN promotes the mTOR/Gli1 pathway, SCD1 promotes the Hippo/YAP pathway, CD36 promotes the DEK/AKT/GSK-3β/β-catenin pathway, FABPs promote the SP1/UCA1 pathway, and HCP5 targets miR-3619-5p to upregulate PPARGC1A, leading to the transactivation of CPT1 by the PGC1α/CEBPB complex. SREBP1: sterol regulatory element-binding protein 1; ACLY: ATP citrate lyase; ACC: acetyl-CoA carboxylase; FASN: fatty acid synthase; SCD1: stearoyl-CoA desaturase 1; MUFAs: monounsaturated FAs; ACSs: acetyl-CoA synthases; H_2_S: hydrogen sulfide; C75: an inhibitor of FASN; A939572: an inhibitor of SCD1; CoA: coenzyme A; Fas: fatty acids; PA: palmitic acid; FAO: fatty acid oxidation; TAC: tricarboxylic acid cycle.

In conclusion, the present impacts and roles of lipid targets in gastric cancer can be summarized (Table 1, Figure 1). When citric acid, a TCA intermediate, is transported from the mitochondria to the cytoplasm by a citrate/isocitrate carrier (CIC), the ACLY in the cytoplasm converts it to acetyl-coenzyme A and oxaloacetate, whereas excessive concentrations of citric acid inhibit the process [112]. MiR-133b and lncRNA FLJ22763 may indirectly regulate this process by affecting the expression and activity of ACLY [38,39]. Acetyl-CoA is then transformed to malonyl-CoA in the presence of ACC. FASN directly employs malonyl-CoA for the synthesis of SFAs, such as PA, because malonyl-CoA is a direct substrate for DNL. Fenofibrate can lower the levels of ACC and FASN expression. By targeting the MTOR/GLI1 signaling pathway, FASN inhibition also lowered gastric cancer development and metastasis [60,109]. The biological function of SCD1 is to generate MUFAs from SFAs, and SCD1 regulates cell stemness via the Hippo/YAP pathway, affecting gastric carcinogenesis, chemoresistance, and metastasis. A939572 treatment reduced the animals’ gastric carcinogenesis, chemo-resistance, and metastasis [70,72]. The primary function of ACSs is to create FA-CoA from intracellular fatty acids, which are then transferred to mitochondria by CPT1, a protein involved in fatty acid oxidation. The FAK and P21 expression levels may be affected by ACSs [46]. At the transcriptional level, SREBP1 regulates the expression of ACLY, ACC, FASN, SCD1, and ACSs, and SREBP1 expression is regulated by HIF-α and Oregano EO [24,27]. CPT1 is a gatekeeper enzyme that mediates the entry of FA-CoA into mitochondria during fatty acid oxidation. HCP5 enhances FAO by activating CPT1 transcription via the miR-3619-5p/AMPK/PGC1/CEBPB axis in gastric cancer [105]. The absorption of exogenous PA into the cytosol is mediated by CD36 and FABPs. In gastric cancer, CD36 mediates DEK/AKT/GSK-3/β-catenin signaling, and CD36 can act as a direct target molecule for hydrogen sulfide. PA-induced GC metastasis is achieved through FABP5/SP1/UCA1 signaling [81,83], and STAT5A increases the tumorigenic capacity of tumor cells by increasing FABP5 expression to regulate fatty acid metabolism [98].

**Figure 2:**
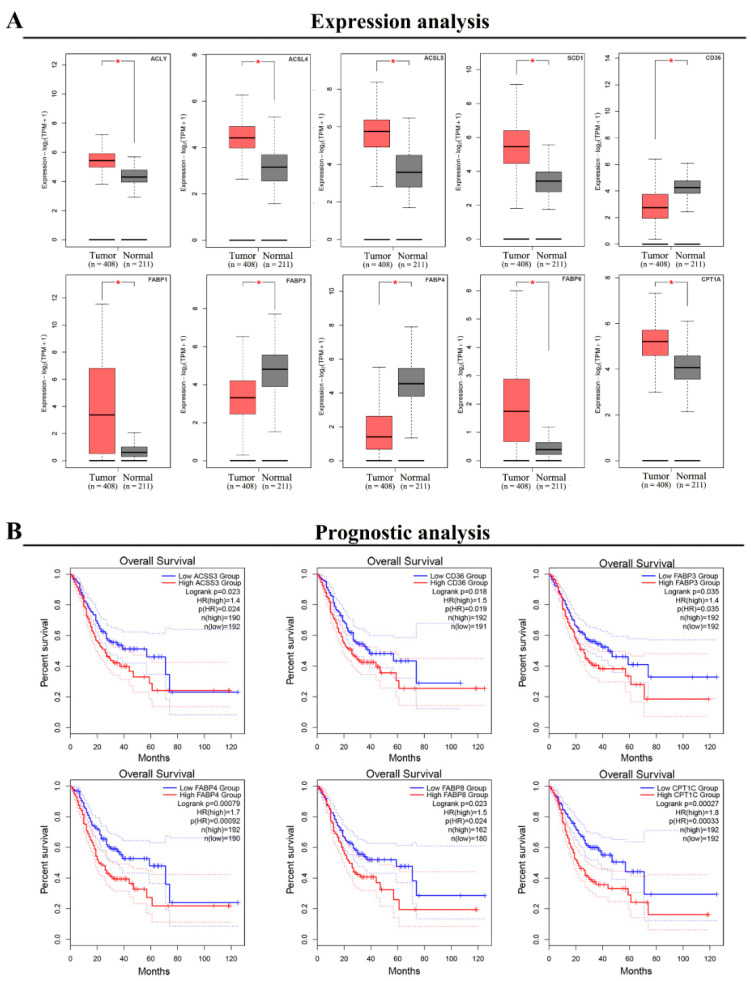
(**A**) Expression of fatty acid metabolism-related genes in GC and paracancerous tissues. (**B**) Correlation between the expression levels of fatty acid metabolism-related genes and the prognosis of GC patients. Red asterisk: *p* < 0.05.

We used The Cancer Genome Atlas and Genotype-Tissue Expression data via GEPIA2 (http://gepia2.cancer-pku.cn/#index, 3 September 2021) to further evaluate the expression of the above genes in GC and paracancerous tissues after examining the research status of fatty acid-related genes and gastric cancer [113]. ACLY, ACLS4, ACSL5, SCD1, FABP1, FABP6, and CPT1A were all found to be substantially expressed in tumor tissue, although CD36, FABP3, and FABP4 were not (Figure 2). We also employed GEPIA2 to examine the relationship between the aforesaid genes’ expression levels and the prognosis of GC patients. We discovered that high levels of ACSS3, CD36, FABP3, FABP4, FABP8, and CPT1C expression were linked to a lower overall survival rate (Figure 2).

## 4. Concluding Remarks

Fatty acid metabolism makes up the majority of lipid metabolism. Fatty acid production and catabolism are also essential for normal cells, and they play a role in tumorigenesis and development. In this study, the association between fatty acid metabolism and stomach cancer is examined. In fatty acid anabolism, SREBP1, ACLY, ACSs, ACC, FASN, and SCD1 enhance the occurrence, progression, and metastasis of gastric cancer, and are expected to be potential prognostic markers. CD36, FABPs, and CPT1, which are fatty acid absorption-related molecules, also contribute to the proliferation of gastric cancer cells. Furthermore, prospective medicines that target fatty acid molecules have the potential to limit gastric cancer cell proliferation, diminish ATP generation, and promote apoptosis. In conclusion, fatty acid metabolism-related compounds may play a new diagnostic or therapeutic function in gastric cancer.

## Figures and Tables

**Table 1:** Expression of fatty acid metabolism-related molecules and their findings and influences in GC.

Molecules	Expression	Findings	Influence
SREBP1	Upregulate [24,25]	Activation of SREBP-1c in GC resulted in upregulation of SCD and FASN and downregulation of ELOVL6. Knockdown of SREBP1c significantly inhibited the proliferation, invasiveness, and migration of GC cells [25]	Tumor promotion [25]
ACLY	Upregulate [33]	Inhibition of ACLY by high-dose sodium citrate reduced the growth of GC in mice [37]	Poor prognosis [33] Tumor promotion [37]
ACSS3	-	ACSS3 knockdown could suppress colony formation under a regular culture, inhibit wound-healing ability under starvation conditions, and increase the basal level of cell death, even more dramatically under starvation conditions [42]	Poor prognosis [42] Tumor promotion [42]
ACSL4	Downregulate [46]	ACSL4 knockdown enhanced cell growth, colony formation, and cell migration in vitro and promoted subcutaneous xenografts’ growth in vivo [46]	Tumor suppressing [46]
ACSL5	-	SiRNA-mediated repression of ACSL5 inhibits the oncogenicity of MKN01 cells [47]	Tumor promotion [47]
pACC	Downregulate [53]	Metformin-induced pACC upregulation resulted in significant inhibition of GC cell proliferation and colony formation [51]	Poor prognosis [51,53] Tumor promotion [51]
FASN	Upregulate [24,58,59,60,62]	The FASN inhibitor C75 or siFASN blocked endogenous fatty acid metabolism in GC and attenuated MACC1 upregulation-induced cell proliferation and chemo-resistance to oxaliplatin to varying degrees [63]	Poor prognosis [59,61] Tumor promotion [60,63] Oxaliplatin resistance [63]
		Inhibition of FASN also inhibited GC proliferation and metastasis by targeting the mTOR/Gli1 signaling pathway [60]	
SCD1	Upregulate [70,71]	SCD1 regulates cell stemness through the Hippo/YAP pathway, which influences gastric carcinogenesis, chemo-resistance, and metastasis [70]	Oxaliplatin resistance [70] Tumor promotion [70,71,72]
		SCD1 promotes the proliferative capacity, migratory capacity, and stemness of GC cells, and SCD1 also has an anti-iron death effect and accelerates the growth of transplanted tumors in mice [71]	
		The mean tumor volume in the A939572-treated group was reduced by nearly 50% relative to vehicle-treated animals [72]	
CD36	Upregulate [77]	CD36 mediates c-Myc-induced DEK transcription in GC cells, then upregulation of DEK enhances GSK-3β/β-catenin signaling [78]	Poor prognosis [78] Tumor promotion [78,80,81]
		Palmitic acid promoted GC metastasis through phosphorylation of AKT and CD36 promoted GC metastasis as a key mediator of AKT/GSK-3β/β-catenin signaling [81]	
FABP4	Downregulate [96] Upregulate [97]	Regulation of FABP4 by a small-molecule FABP4 inhibitor or siFABP4 restores primary cilia to inhibit the proliferation and migration of GCs, thus exhibiting potential anticancer effects [94]	Tumor promotion [94] Poor prognosis [94,96]
FABP5	Upregulate [97]	Silencing of the FABP5 gene attenuated the invasiveness of GC cells, prevented cell proliferation, and stalled the cell cycle in the G0/G1 phase, leading to a significant increase in apoptosis [95,99]	Tumor promotion [95,97,98,99,101]
		PA enters GC cells, promotes the nuclear transport of FABP5, which then increases the GC nuclear protein levels of SP1 and PA-induced GC metastasis via FABP5/SP1/UCA1 signaling, contributing efficient prevention and therapeutic strategies for GC [100]	
CPT1	Upregulate [106,107]	CPT1A overexpression activates fatty acid oxidation in GC cells by increasing the NADP/NADPH ratio and thus increases the proliferation, invasion, and epithelial–mesenchymal transition (EMT) of GC cells [106]	Poor prognosis [106,111] Enhanced tumorigenesis [106,108,110]
		CPT1A succinylates LDHA on K222, which thereby reduces the binding and inhibits the degradation of LDHA and promotes GC invasion and proliferation [108]	
		The CPT1 inhibitor perhexiline and oxaliplatin synergistically inhibit tumor xenograft progression, suggesting that CPT1-mediated fatty acid translocation and further fatty acid oxidation may be associated with oxaliplatin resistance [110]	

## Data Availability

Not applicable.

## Abbreviations

GC: gastric cancer; DNL, de novo lipogenesis; FAs, fatty acids; acetyl-CoA, acetyl-coenzyme A; SFAs, saturated fatty acids; OA, oleic acid; MUFA, monounsaturated fatty acid; AA, arachidonic; EPA, eicosapentaenoic acid; DHA, docosahexaenoic acid; PUFAs, polyunsaturated fatty acids; ROS, reactive oxygen species; SREBP1, sterol regulatory element-binding protein 1; HIF-1α, stimulation of hypoxia-inducible factor 1α; GPX4, glutathione peroxidase 4; HMGCR, 3-hydroxy-3-methylglutaryl-CoA reductase; ACLY, ATP citrate lyase; TCA, tricarboxylic acid cycle; ACSs, acetyl-CoA synthases; FA-CoA, fatty acyl-CoA; DFS, disease-free survival; OS, overall survival; ACC, acetyl-CoA carboxylase; pACC, phosphorylated acetyl-CoA carboxylase; ICA, immune cytolytic activity; FASN, fatty acid synthase; mHER2, membranous HER2; SCD1, stearoyl-CoA desaturase 1; PA, palmitic acid; MUFAs, monounsaturated fatty acids; H2S, hydrogen sulfide; FABPs, fatty acid-binding proteins; STAD, stomach adenocarcinoma; TRM, tissue-resident memory T cells; CPT1, carnitine palmitoyltransferase 1; FAO, fatty acid oxidation; EMT, epithelial–mesenchymal transition; CIC, citrate/isocitrate carrier.
